# Should eloquence be taught as part of the undergraduate medical curriculum?

**DOI:** 10.15694/mep.2021.000087.1

**Published:** 2021-04-07

**Authors:** Lauren Kennedy, Laila Cunin

**Affiliations:** 1 East Kent University Hospitals Foundation NHS Trust; 2 East Sussex Healthcare NHS Trust

**Keywords:** eloquence, medical humanities, rhetoric, curriculum, public speaking

## Abstract

This article was migrated. The article was marked as recommended.

The integration of the humanities into the medical curriculum over the past two decades has been widely adopted in the stream of communication usually within the realms of doctor-patient relationship. However, its integration within medical curriculum is inconsistent, and may only be present as an optional component in certain selected modules. The study of eloquence within medicine has not been described previously, we propose that its inclusion into the medical curricula will increase equality and diversity in medical training. We aim to debate the roles of medical humanities and the integration of eloquence into the medical curriculum. Integration of eloquence into the medical curriculum with the aim of developing written prose and oration could improve our interprofessional communication and bridge the gap for those from a wider social background accessing medicine.

## Introduction

Good communication skills are pivotal for a successful doctor-patient relationship ( Ong *et al.,* 1995) this forms a core component of medical curricula and is essential to Good Medical Practice; outlined by the General Medical Council ( General Medical Council, 2013). The GMC specifies that a doctor should have the ability to communicate effectively to patients, those close to patients and to colleagues. It is within this guidance that the GMC includes communication with colleagues as an important tenet, to ensure we work to best serve the patient’s interest. The domain of “Communication partnership and teamwork” includes the subsection teaching and training as part of that duty we should teach students and junior doctors. By extension of this principle, we as scientists are expected to effectively convey our results by means of oration, e.g. In a conference auditorium, or via publication as scientific prose with the hope of acceptance to high impact journals, thus communication skills are imperative to facilitate distribution of key information. However, the way in which we present our concepts can influence their acceptability and overall impact to practice, therefore the argument should be made for teaching eloquence in presentation as part of the medical curriculum. Integration of the arts and humanities with the sciences will transfer this subject from its current position within the hidden curriculum under the guise of professional skills and personal development into the formal curriculum ( Suchman *et al.,* 2004). Historically there is a perception that medical training and higher-level training and employment is more accessible to affluent families and those educated in the Independent schools’ system, where eloquence is taught during school years. Incorporating this into current curricula may enhance diversity and accessibility of training and academia for future generations of doctors.

## Current place of humanities within the medical curriculum

As part of the GMC series “Tomorrow’s Doctors” ( General Medical Council, 1993) recommended that the subjects of arts and humanities be offered as part of special study modules as part of the medical undergraduate course. The term “medical humanities” was actually coined at least five decades prior to this however integration within the undergraduate curricula has only occurred early this Century ( Hurwitz and Dakin, 2009). Downie ( Downie, 2016) categorised the humanities as having 3 separate functions: the supplementary function, the critical function and personal and professional development.

The supplementary function is to aid the learning and discussion of ethics and communication through the discussion and analyses of literature, theatre and film. He suggests that these discussions go beyond the 4 ethical principles of Beauchamp and Childress ( Beauchamp and Childress, 2001) and therefore better address humility, morality and go beyond the technicality of communication skills; into the creative world of human emotions and responses. He proposes that communication skills as a subject should surpass what is currently taught in the medical school curricula.

The assertation that humanities impact communication skills has been evidenced by a recent observational study from the USA: those who majored in the humanities and social sciences as their pre-med performed significantly better in their communication and interpersonal skills examination that those with natural science majors ( Hirshfield, Yudkowsky and Park, 2019). The theory of communication skills training, postulates that communication as a skill is transactional ( Piemonte, 2017) and our undergraduate curricula work along this basis. A literature review in 2018 ( Sanson-Fisher *et al.,* 2018) looked at the methodological quality of research in communication skills, upon its review of 243 publications the authors reported that the majority of these were descriptive and not evidence based. In order to progress practice beyond restrictive models such as Situation/Background/Assessment and Recommendation (SBAR) or the Calgary-Cambridge guide ( Brindley *et al.,* 2014), a combined approach with increasing scope for research and integration with the current medical school curricula will be imperative. It is undeniable that current teaching is a useful adjunct to training and can be useful starting points or *aide memoires* in the education of communication.

The critical function of the medical humanities is to offer the medical community a philosophical view of medical, public health and professional issues in order to improve good clinical judgement. By challenging students through philosophers’ quandaries, they can reflect and embark on a journey of self-development, which will be beneficial both to their long-term wellbeing and that of their patients and patients’ families. The emergence of “critical medical humanities” ( Viney, Callard and Woods, 2015) as a subject calls for collaboration across specialities and disciplines and the medical humanities in its critique of healthcare ( Whitehead, 2016). By challenging the ways in which we approach healthcare and the everyday “norms” we can improve patient care.

The use of the arts can be a contributor to both personal and professional development of a practitioner by facilitating a balance of scientific fact and the compassion and care towards patients. With the concentration on the biomedical approach to treatment of disease it is thought that this can compromise clinical empathy towards the patients ( Garden, 2007). Thus, the assumption is that the personal development goes beyond being a good practitioner and towards being an overall better human.

To summarise the use of humanities in medical education the Edinburgh Companion ( Whitehead, 2016) suggests the five E’s approach: ethics, education and experience and the associated engagement and empathy. The use of humanities in the medical curriculum is predominantly established in its usefulness in the communication between doctor and patient; their relationship and ethical considerations that may be encountered. What I would like to suggest is to extend the humanities programme and use it as a tool to teach eloquence to inspire us, enhance our communication with colleagues in the scientific arena such as at conferences and through publication; enhance our communication with the public via media and educational resources and therefore to integrate the biomedical and humanities of our curricula as opposed to them being housed separately.

## Defining eloquence and rhetoric

Eloquence by definition is the fluency and persuasion of verbal or written presentation ( Stevenson, 2010). Eloquent speakers such as Winston Churchill, Martin Luther King and Barack Obama have made huge impact through their use and presentation of the English language, their speeches are not only unforgettable but influential. Eloquent writers create memorable works, many of us can quote the famous phrases such as William Shakespeare’s Hamlet “to be or not to be” ( Shakespeare, 2014); Cormac McCarthy’s The Road “You forget what you want to remember, and you remember what you want to forget” ( McCarthy, 2007) and Oscar Wilde’s The Picture of Dorian Gray “Nowadays people know the price of everything and the value of nothing” ( Wilde and Page, 1998). The commonality with these writers is that their use of language is powerful and noteworthy. I am not suggesting that all medics should study English literature, but we can learn from the literature and improve the communication of our knowledge, our skills and our results with each other and the general population. Rhetoric which is thought to originate from the ancient Greek philosophers, such as Plato and Aristotle, is defined as the art of persuasion and effectiveness in speech and writing. The study of rhetoric focuses upon composition techniques to aid its impact, however some view rhetoric with cynicism as it need not be a moral form of persuasion. Overall, books and papers on the subject of eloquence call upon rhetorical techniques to create eloquent work which can be key in highlighting important matters in health and social care.

Eloquence, rhetoric, public speaking and linguistic resources are plentiful. From novels to media, for example, there are a plethora of TED talks and YouTube videos offering tips on how to structure a great speech and advice on language, from syntax to phonology ( TEDx, 2016). The language of rhetoric was commonly placed in the national curriculum historically, however it is no longer accessible for those educated in the public sector and is really only accessed by those studying beyond the statutory national curriculum learning either Latin or Greek. By enabling all medical students to access this learning, not just those from more elite backgrounds, it will be easier to expand their lexicon and facilitate them to orate and produce publications that meet the high standards required for transfer of a meaningful message to all peers and patients. This will mean our practice of medicine is as diverse as our patient population and that we can all access learning from sources that would not previously have been the Orators most heard.

There are many rules of language we can use such as: the rule of 3 and pathos, ethos and logos. However, will hours of reading and listening to these resources result in a good speech if the pace and phonology is incorrect? If communication skills cannot be taught didactically this implies a need for this teaching to be interactive with reflection and feedback pivotal to progression as it is in our other communication skills training. An editorial written by Campbell ( Campbell, 2018) argues for the promoted use of a separate field, rhetoric of health and medicine, to engage and produce humanistic clinicians through simulation. She also suggests that working with the interprofessional teams and the medical humanities we can enhance the doctor-patient relationship and health literacy.

## Eloquence, the arts and current curricula

Prose

In order to ascertain if education of eloquence is currently being included in curricula Pubmed, EBSCO and google scholar searches were performed. There was no published literature in the field of eloquence in the medical education curricula. Existing literature on the subject of humanities largely consist of editorials representing personal views ( Ousager and Johannessen, 2010) with few attempts to evidence the long term impact of such curricula. Relevant to the topic of eloquence were 2 articles specific to the art of writing a research abstracts ( Papanas *et al.,* 2012; Evans, 1994) which address preparation and structure with recommendations to avoid vague statements, however, they do not refer to linguistics.

There are several articles with recommendations for writing medical case reports; many fail to address grammar, syntax and style ( Florek and Dellavalle, 2016; Rison, 2013) and concentrate on why to write and how to structure the paper. An analysis of historical medical case report writing published in the Journal of English Linguistics ( Taavitsainen and Pahta, 2000) documents the changes in our writing from the narrative accounts of the 19 ^th^ and early 20 ^th^ century to our detached 3 ^rd^ person accounts of today. It suggests that scientific discourse has resulted in the demise to semiotic writing and therefore training in eloquence would be futile for the intention of enabling publication of case reports. An editorial series in the medical education journal: ‘When I say...” ( Walsh and Eva, 2013) asked readers to submit interesting narrative pieces on the definition of a concept, for example problem-based learning with the attempt to merge the worlds of creativity and etymology. The need for rhetoric in protocol writing was called for in 2009 in a scathing article of clinical written work focussing specifically on protocols and guidelines ( Bell, Walch and Katz, 2000).

Oration and Linguistics

A novel way of integrating public speaking into the curriculum was described by Rieger *et al.* in 2017 ( Rieger, Aggarwal and Cameron, 2017) as the introduction of scientific elevator speeches: a programme delivered via two 90 minute workshops, the first introduced content and structure of an elevator speech and the second workshop on style of delivery. 10 participants were chosen as finalists to introduce and deliver their research project in 90 seconds in a public speaking competition. Participants viewed this as a valuable project which not only allowed for styling and presentation skills, but also helping them gain overall clarity and understanding of their research work, enhanced their critical thinking and their professional development. In another university department, educationalists incorporated public speaking programmes for lecturers which resulted in improved lecturer confidence and implied an increase in the impact of their talks by the subsequently improved student results (statistically significant on *x* ^2^ testing) ( Mowbray and Perry, 2015), they also suggest that having immediate feedback on lecture quality resulted in greater proficiency of public speaking and its performance ( King, Young and Behnke, 2000).

## Challenges for the future

English is accepted as the universal language of the sciences, for conferences and international publication one must submit a well written abstract or article. This can be challenging to many graduates but especially for those in whom English is not their first language ( Kourilova, 1979). The introduction of eloquence may be a welcome addition to both the undergraduate and postgraduate curriculum and may enable our international community to have better access and perhaps lead to higher impact publications. In addition, language and linguistic ability vary in communication skills education and reflective writing pieces in those from different socioeconomic or cultural groups ( Boromisza-Habashi, Hughes and Malkowski, 2016) and perhaps by introducing eloquence to medicine will attempt to bridge this existing gap between non-selective state school educated and independent school learners enhancing diversity.

Public speaking is more than words, it requires powerful use of non-verbal cues such as deep breathing, the use of pauses and silence as well as body language. The art of public speaking will not only serve those speaking at local, regional and national meetings but to those speaking at international conferences and for those educating the public via media resources from newspapers to social media and television.

With regards to eloquence in speaking I propose enlisting the help of the humanities, rhetoric in health and medicine, linguistics, English scholars and drama/media/arts teams who already seem to have integrated into curricula, in variable quantities. Creating a new and integrated, interprofessional curriculum with the aforementioned faculties, will entrench good communication skills beyond those already existing in the curriculum; such as the use of role-play in breaking bad news, handling conflict and opening dialogue into biomedical ethics ( McCullough, 2012). Although the value of communication skills training through medical school cannot be contested and is an essential component of GMC’s Good Medical Practice and Tomorrow’s Doctors, studies have indicated that communication skills training and its value is met with scepticism and negativity by medical students ( Moral *et al.,* 2019; Wright *et al.,* 2006; Rees and Sheard, 2002). Prolonging this part of the course may not be welcomed, traditionally this aspect of medical training has been devalued and deemed to not be academic, enabling engagement with academia through writing with eloquence and facilitating students to develop their academic writing skills may address this concern. Perhaps this additional content will engage those sceptics? Another option may be to introduce it as a selective module separate to gauge interest in the subject and research its value as a topic in medicine.

Written eloquence would perhaps warrant a different approach and be integrated into the research skills. There are many courses, publications and lectures available to undergraduate and postgraduates in “How to write a paper”. However, these courses are currently only available as paid short courses and have limited capacity to tailor feedback on an individual level. If the aim is solely to publish a paper in order to “Publish or perish” ( Garfield, 1996; Moral *et al.,* 2019), this allows the generation of authors who perhaps do not regard to having good prose in their writing as necessary. In addition, with the general abolition of written examination in medical schools in favour of multiple choice there is little written work that allows for feedback on students’ English writing capabilities nor encouragement to become proficient in writing eloquently and hence to communicate effectively in the written context. I do not propose that we revert back to this method of examination but instead exercise the argument for more exposure to written works and analyses. Again, I propose the integration of the aforementioned interprofessional fields into our research curricula to aid our written rhetoric and eloquence in addition to our knowledge and understanding of the scientific content of health literature.

## Conclusion

The marrying of the humanities with eloquence in oratory and literature can only be beneficial for medical students and junior doctors as part of their lifelong learning. The extension of the communication skills programmes will allow the humanities to engage the student in performance of oral presentation and in involving the arts, namely linguists and scholars of literature, to help us in our written presentation of our work. Both of these collaborations would help us integrate the scientific and arts curricula.

The use of humanities as adjuncts to cover the subjects of morality and ethics are hard to assess and although their impact on students can be profound, their place within the medical curricula has struggled to gain widespread and universal acceptance. The use of the humanities, and more specifically eloquence, in cases of oratory and written presentation can be assessed, examined and quantified and the adage that this may result in research opportunities which will appeal to many doctor’s scientific nature.

With its introduction to a medical curriculum, we should address the research hypothesis of whether the training in eloquence will impact our oration and prose. Working with our colleagues in the arts and humanities, English literature and linguistics to help us integrate eloquence into our curriculum. In particular, introducing the study of syntax, rhetoric and discourse analysis I hope will encourage lateral thinkers and who will have greater clarity in their scientific expression as a result of a truly interprofessional effort for healthcare and personal development.

## Take Home Messages

The teaching of eloquence could offer:

• Improved oration and prose
• Improved communication skills
• A method of professional and personal development
• A way of breaking the barriers to equality in education

## Notes On Contributors

Lauren Kennedy is a ST8 in Upper GI & General Surgery at East Kent Hospitals University NHS Foundation Trust with an interest in both undergraduate and postgraduate medical education, previously a clinical teaching fellow, she has lead postgraduate simulation courses as well as implemented her regional higher surgical teaching programme. ORCiD: https://orcid.org/0000-0002-0703-8197

Laila Cunin is a ST7 in Colorectal & General Surgery at East Sussex Healthcare NHS Trust, she is involved in undergraduate, postgraduate and multidisciplinary education and is currently leading the regional core surgical training teaching programme. ORCiD: https://orcid.org/0000-0002-5686-2107
