# Supplementary material for: Should eloquence be taught as part of the undergraduate medical curriculum?
Source: MedEdPublish (2016). 2021 Apr 7;10:87. [Version 1] doi: 10.15694/mep.2021.000087.1 (PMC10939647; doi:10.15694/mep.2021.000087.1)
Supplement: Peer review history

# Reviewer report: Reviewer response for version 1

**DOI:** 10.21956/mep.18968.r27163

**Published:** 2021-04-28

**Authors:** Balakrishnan Nair

**Affiliations:** 1 Centre for Medical Professional Development and University of Newcastle

This review has been migrated. The reviewer awarded 3 stars out of 5 Doctors should have good communication skills which include oral( including body language) and written skills . So this article on eloquence is very appropriate and the authors have done a good job of articulating this . They have given some good examples from Shakespeare to Oscar Wilde. Another good example would have been from Sir William Osler like “The good physician treats the disease; the great physician treats the patient who has the disease”. His text book written more than a century ago is still a master piece in writing The article could have been better with some more editing . Some of the sentences are too long. Whilst there are two authors, many statements are state “ I would like to suggest “, “ I am not suggesting “ I propose “ etc . In spite of these , I enjoyed reading this and agree with the authors

Reviewer Expertise:

NA

No decision status is available

# Reviewer report: Reviewer response for version 1

**DOI:** 10.21956/mep.18968.r27159

**Published:** 2021-04-28

**Authors:** Mohamed Al-Eraky

**Affiliations:** 1 University of Dammam

This review has been migrated. The reviewer awarded 4 stars out of 5 Thank you. I truly enjoyed reading this perspective and I think the debate will continue about expanding medical curricula to accommodate, subjects like: eloquence, the E’s approach (of Edinburgh) and many others. The question is not only about teaching, but also assessment that has to be relevant to clinical practice. The argument is usually about feasibility, efficiency, readiness of students and competencies of teachers. Some would argue that future physicians ‘need’ to learn everything related to: communication, technology, law and even business administration and the foundations for entrepreneurship to manage their future private clinics. Yet, we may need to acknowledge the limited time available in undergraduate medical education and decide what’s really vital, essential, must learn, good to learn or nice to learn. Thanks for this reflective article. Good luck!

Reviewer Expertise:

NA

No decision status is available

# Reviewer report: Reviewer response for version 1

**DOI:** 10.21956/mep.18968.r27160

**Published:** 2021-04-27

**Authors:** Tharin Phenwan

**Affiliations:** 1 University of Dundee

This review has been migrated. The reviewer awarded 4 stars out of 5 This is an interesting, thought-provoking work. I agreed with your suggestions and also felt a bit surprised with the dearth of literature out there. The potential challenge would be how to integrate this novel suggestions with the current situation where we have chronic curriculomegaly. Perhaps it will be practical to do this in a pilot group with your setting to see its feasibility?Also, I have two more questions:-The introduction part mentioned that there is a perception that medical training is more accessible to affluent families due to various factors. Is this still the case currently?-When did you perform your search?This should be read by those with an interest in Behavioural and Social Sciences and Professionalism

Reviewer Expertise:

NA

No decision status is available

# Reviewer report: Reviewer response for version 1

**DOI:** 10.21956/mep.18968.r27162

**Published:** 2021-04-16

**Authors:** Leila Niemi-Murola

**Affiliations:** 1 University of Helsinki

This review has been migrated. The reviewer awarded 5 stars out of 5 This is an interesting article focusing on teaching of eloquence as a part of undergraduate curriculum. The word itself was new to me, but according to my dictionary it is closely related to rhetoric. As the authors point out, good communication skills are for a physician. This is not just what we say and how we present it, but the way we present it, i.e. how we deliver our message. The authors speak very convincingly in the favor in including eloquence in undergraduate curriculum. I fully agree, and I can see the potential barrier. In my country, the undergraduate program consists of 360 credits (one credit is equivalent to 27 hours of work). If we add something, we should also remove something. We already have a communication skills course, which is fully packed. However, there are always elective and voluntary studies, and we could pilot this kind of course as an elective one.

Reviewer Expertise:

NA

No decision status is available

# Reviewer report: Reviewer response for version 1

**DOI:** 10.21956/mep.18968.r27161

**Published:** 2021-04-07

**Authors:** Virginia Randall

**Affiliations:** 1 Uniformed Services University of the Health Sciences

This review has been migrated. The reviewer awarded 4 stars out of 5 What a "breath of fresh air." Eloquent writing aside, this was an enjoyable article to read and contemplate, and opened up some new ways of thinking about teaching (especially through reviewing some of the references.) It also demands that we, as educators, model eloquence in our interactions and writing. I appreciate that some students who complete the humanities in medicine classes will feel empowered to read non-scientific literature for pleasure throughout their careers, which may lead to medical care being more equitable.

Reviewer Expertise:

NA

No decision status is available
